# Up-regulation of S100A16 expression promotes epithelial-mesenchymal transition via Notch1 pathway in breast cancer

**DOI:** 10.1186/s12929-014-0097-8

**Published:** 2014-10-07

**Authors:** Wenbin Zhou, Hong Pan, Tiansong Xia, Jinqiu Xue, Lin Cheng, Ping Fan, Yifen Zhang, Weidong Zhu, Yi Xue, Xiaoan Liu, Qiang Ding, Yun Liu, Shui Wang

**Affiliations:** Department of Breast Surgery, The First Affiliated Hospital with Nanjing Medical University, 300 Guangzhou Road, 210029 Nanjing, China; Department of Oncology, Lombardi Comprehensive Cancer Center, Georgetown University, Washington, DC 20057 USA; Department of Pathology, Nanjing Drum Tower Hospital, Nanjing University Medical School, 321 Zhongshan Road, 210008 Nanjing, China; Department of Urology, Zhongda Hospital Affiliated to Southeast University, Nanjing, 210008 China; Department of Geratology, The First Affiliated Hospital with Nanjing Medical University, 300 Guangzhou Road, 210029 Nanjing, China

**Keywords:** Breast cancer, S100A16, EMT, Notch1, MCF-7

## Abstract

**Background:**

Our previous studies demonstrated that S100A16 promotes adipogenesis and is involved in weight gain attenuation induced by dietary calcium. Till now, the function of S100A16 in the breast cancer remains to be elucidated.

**Results:**

In this study, we observed that S100A16 was expressed in higher levels in human breast cancer tissues compared with paired adjacent non-cancerous tissues. Further examination showed that overexpression of S100A16 in MCF-7 cells could increase cell proliferation and colony formation. One major mechanistic change was that S100A16 was able to up-regulate the transcription factors Notch1, ZEB1, and ZEB2, which had the capacities to directly repress the expression of epithelial markers E-cadherin and β-catenin but increase mesenchymal markers N-cadherin and vimentin, a characterized phenotype of epithelial-mensenchymal transition (EMT). In addition to display with morphologic change, migration and invasion were increased in S100A16 over-expressed MCF-7 cells. Importantly, knockdown of Notch1 by specific siRNA could reverse the EMT induced by S100A16 overexpression, which confirmed that Notch1 played a critical role in the process of EMT induced by S100A16.

**Conclusions:**

All together, our data indicated that S100A16 had a potential function to regulate some embryonic transcription factors to promote EMT in breast cancer cells which may be an important target site for the therapy of breast cancer.

**Electronic supplementary material:**

The online version of this article (doi:10.1186/s12929-014-0097-8) contains supplementary material, which is available to authorized users.

## Background

Breast cancer is a worldwide malignant disease. Although disease-free survival and overall survival have been significantly improved, there are still some parts of patients diagnosed with metastases after systemic therapies [1]. The poor survival of advanced breast cancer is related to the lack of effective therapies, which can be attributed to poor understanding of the molecular mechanisms underlying the progression of disease toward invasion and metastasis [2,3]. Up to now, many molecular markers have been found, such as estrogen receptor (ER), progesterone receptor (PR) and HER2. ER and/or PR positive are considered as hormone receptor positive (HR+), which is a predictor of endocrine therapy and good clinical outcome. HER2 is an indicator of targeted therapy and a predictor of poor clinical outcome. However, there is huge desire to improve the survival of advanced breast cancer. Therefore, it is important to identify new predictive biomarkers, especially those indicators for invasiveness of the disease.

S100A16 is a novel low-affinity calcium-binding protein of the EF-hand superfamily [4–6]. Our previous studies suggest that S100A16 promotes adipogenesis and is involved in weight gain attenuation induced by dietary calcium [7,8]. Importantly, S100A16 is a ubiquitously expressed protein, which is up-regulated in various malignant tumors [5,9]. However, the physiological functions of S100A16 in malignant tumors are still undefined.

The epithelial-mesenchymal transition (EMT) plays a crucial role in the formation and differentiation of multiple tissues. Importantly, it is clear that EMT is involved in metastatic events in cancer and highly relevant to tumor progression [10]. Notch signaling pathway affects cancer patient survival, especially for Notch1 and Notch4 receptors [11]. Activation of the Notch signaling pathway participates in EMT induction [12,13].

All of these findings imply that S100A16 may be involved in the progression of breast cancer. To investigate the function of S100A16 in breast cancer, we first documented here that S100A16 was over-expressed in breast cancer tumors. Further examination demonstrated that up-regulation of S100A16 promoted EMT via Notch1 pathway in breast cancer cell line MCF-7. Our results suggested that S100A16 may be involved in the progress of breast cancer.

## Methods

### RNA extraction, reverse transcription and quantitative RT-PCR

The Trizol reagent (Invitrogen) was used to isolate total RNA following manufacturer’s instructions. Then RNA was used for cDNA synthesis using a reverse transcriptase reaction kit (TaKaRa). Quantitative RT-PCR for β-actin and other genes was performed for every cDNA sample. All PCR reactions were performed using the fluorescent SYBR Green I methodology. Real-time quantitative PCR was performed with SYBR Premix Ex Taq (TaKaRa) according to the manufacturer’s instructions. β-actin was used as a loading control.

S100A16 expression was measured in 20 breast cancer tissue samples compared with paired adjacent non-cancerous tissue using qRT-PCR. The paired non-cancerous tissues were adjacent non- cancerous tissue, which were at least 5 cm away from the cancerous tissue, which were normal “histologically”. This study was approved by the ethics commit of the First Affiliated Hospital with Nanjing Medical University. All patients provided written informed consent for their clinical information to be reviewed by us. And it was in compliance with the Helsinki Declaration.

### Cell culture

The human breast cancer cell lines MCF-7, T47D, BT474, ZR-75-1, MDA-MB-231, BT549, MCF10A, 184A1 and 184B5 were obtained from the American Tissue Culture Collection (ATCC), and cultured in complete medium (DMEM, IMEM or 1640 supplemented with 10% fetal bovine serum) in a 5% CO_2_ 37°C incubator.

### Plasmid construction and lentivirus packaging

S100A16 gene was cloned by RT-PCR from RNA of human MCF-7 cells and cloned into the pLV-GFP vector (a gift from Dr. Beicheng Sun, Nanjing Medical University, China) using BamHIand MluI sites, named pLV-S100A16. Plasmid sequencing was performed by Invitrogen Company with a 3730 DNA analyzer. Primer sequences for S100A16: Forward: TTGGATCCGGAGATGTCAGACTGCTACAC, Reverse: TTACGCGTAAAGGGGTCTCTAGCTGCTG.

Recombinant lentivirus was generated from 293 T cells using calcium phosphate precipitation. The levtivirus was then transduced into MCF-7 and T47D cells mediated by polybrene (8 μg/ml). Plain pLV-GFP lentivirus infected MCF-7 and T47D cells were used as the control.

### Colony formation assay

Cells used for colony formation analysis were seeded into 6-well plates (1000 cells/ml) and cultured normally for two weeks. The colonies were fixed in paraform and stained with Giemsa after washed with PBS twice. The number of colonies was counted.

### MTT assay

MTT assay was applied to assess cell proliferation as described previously [14]. Cell proliferation was compared at five time point (1, 2, 3, 4, and 5 days) after the cells were seeded. The absorbance of the samples was measured at 490 nm on a scanning multi-well spectrophotometer. Each experiment was repeated three times.

### Cell migration and invasion assay

In vitro cell migration and invasion assays were performed as described previously [15]. Cells growing in the log phase were trypsinized, re-suspended in serum-free medium, and seeded into Boyden chamebers (8 μm pore size with polycarbonate membrane). The chambers were then inserted into transwell apparatus (Costar, Cambridge, MA, USA). The chambers were coated with Matrigel (BD Biosciences, San Jose, USA) when cell invasion assay was done. And the chambers were coated without Matrigel when cell migration assay was done. Medium with 10% FBS (600 μl) was added to the lower chamber. After incubation of 24 h, cells on the top surface of the insert were removed by wiping with cotton swab. Cells that migrated to the bottom surface of the insert were stained in 0.1% crystal violet for 30 min, rinsed in PBS and then subjected to microscopic inspection. Images of three random fields (10×) were captured from each membrane, and the number of migratory or invasive cells was counted. Triplicate assays were used for each experiment.

### Notch1 small interfering RNA transfection

Notch1 siRNA were purchased from GenePharma (Shanghai, China). MCF7-S10016 cells were transfected with Notch1 siRNA as described previously [14]. The sequences of Notch1 were GUC CAG GAA ACA ACU GCA ATT (sense) and UUG CAG UUG UUU CCU GGA CTT (antisense), respectively. The sequences of negative control were UUC UCC GAA CGU GUC ACG UTT (sense) and ACG UGA CAC GUU CGG AGA ATT (antisense), respectively.

### Western blot analysis

Western blot analysis was performed as described previously [14]. Monoclonal anti-S100A16 (R&D), ZEB1 (Santa Cruz), ZEB2, Notch1 (Abcam), β-Catenin, N-Cadherin, E-cadherin, Vimentin were purchased. GAPDH (Santa Cruz) was used to as loading control. The intensity of the bands was determined by using densitometric analysis. All experiments were performed three times.

### Immunohistochemistry and Fluorescence in situ hybridization

Breast tissue samples were obtained at the time of surgery. Immunohistochemistry (IHC) analyses were performed on 4 μm, formalin-fixed, paraffin-embedded slides from breast cancer tissues and matched non-cancerous tissues. Paraffin-embedded tissue sections were deparaffinized, rehydrated, rinsed, and immersed in 10 mM sodium citrate (pH 6.0) for antigen retrieval under high pressure in a pressure cooker for 3 minutes. After treated with in methanol containing 3% hydrogen peroxide for 10 min to block endogenous peroxidase activity, the slides were incubated with primary antibody for 1 hour at 37°C. After washing, sequential incubations were performed with horseradish peroxidase (HRP) conjugated antibodies (Invitrogen) for 30 min at room temperature. The stain was visualized using DAB Plus (Dako) and hematoxylin counterstain. The expressions of S100A16, ER, PR, HER2 were all detected by IHC.

A Fluorescence in situ hybridization (FISH) test for HER2 gene amplification was routinely ordered when HER2 was IHC 2+. FISH was performed using the PathVysion HER2 DNA FISH Kit (Vysis Inc, Downers Grove, IL), according to the manufacturer’s instructions.

### Immunofluorescence

Cells were seeded in a 6-well culture plate. The cells were immunofluorescence-labeled according to the manufacturer’s instructions. Cells were incubated with a blocking buffer for 1 hour to suppress non-specific binding after washing and fixing. And then, cells were incubated with primary antibody (ZEB1, ZEB2, β-Catenin, N-Cadherin, E-cadherin, Vimentin) at 4°C overnight, followed by incubation with a secondary antibody for 1 hour. Cells were further mounted with DAPI (4′, 6-diamidino-2-phenylindole) for 5 minutes and analyzed using fluorescence microscopy.

### Luciferase reporter assay

We analyzed the putative promoter region of Notch1 (−1998 to +76) using a luciferase reporter assay. The Notch1 promoter was cloned by PCR from genomic DNA of human MDA-MB-231 cells. PCR-based cloning was used to generate different segments (−483 to +76, −998 to +76, −1496 to +76, and −1998 to +76) of the Notch-1 gene promoter. Then the amplified products were ligated into the pGL3 basic vector using NheI and XhoI sites (New England Biolab, UK), respectively, named pGL3-Notch1-I, pGL3-Notch1-II, pGL3-Notch1-III, and pGL3-Notch1-IV. Plasmid sequencing was performed by Invitrogen Company (China) with a 3730 DNA analyzer. Primer sequences for Notch-1 promoter cloning:

- Forward 1:5′-ACTTGCTAGCGAGAAGTAGTCCCAGGCGC-3′,
- Forward 2:5′-CCGGGCTAGCGGAGGACGGTGACCGAG-3′,
- Forward 3:5′-GGCTGCTAGCAGAGTGCTCAGCATTTGGC-3′,
- Forward 4:5′-ACCAGCTAGCACCCCCTATCCAGGGATC-3′,
- Reverse: 5′-TGGGCTCGAGCCTACCTCGTGCGGCG −3′.

For the S100A16 responsive promoter activity, S100A16-transduced MCF-7 cells, MCF7-S100A16 cells were chosen to analyze the putative promoter region of Notch1 (−1998 to +76) using dual luciferase reporter assay. MCF7-S100A16 cells were seeded into 6-well culture plates 1 day prior to transient transfection. Plasmids pGL3-Notch1-I, pGL3-Notch1-α, pGL3-Notch1-β, and pGL3-Notch1-Ις were respectively co-transfected with the Renilla luciferase expression plasmid into MCF7-S100A16 cells. After 48 h transfection, cells were collected and luciferase activity was measured using the Dual Luciferase Reporter Assay System (Promega, USA) according to the manufacturer’s instructions. Then, the cells were harvested and processed for dual-luciferase reporter activity, in which the firefly luciferase activity was normalized by renilla luciferase activity.

### Statistical analysis

All experiments in this study were repeated in triplicate. Paired *t*-test was applied to calculate the statistical significance of the expression levels of S100A16 mRNA in breast cancer tissue and adjacent non-cancerous tissue. Student *t* test was applied to calculate the statistical significance of other experimental results. A significant difference was concluded for *P* < 0.05.

## Results

### S100A16 was overexpressed in human breast cancer tissues

**Figure 1:**
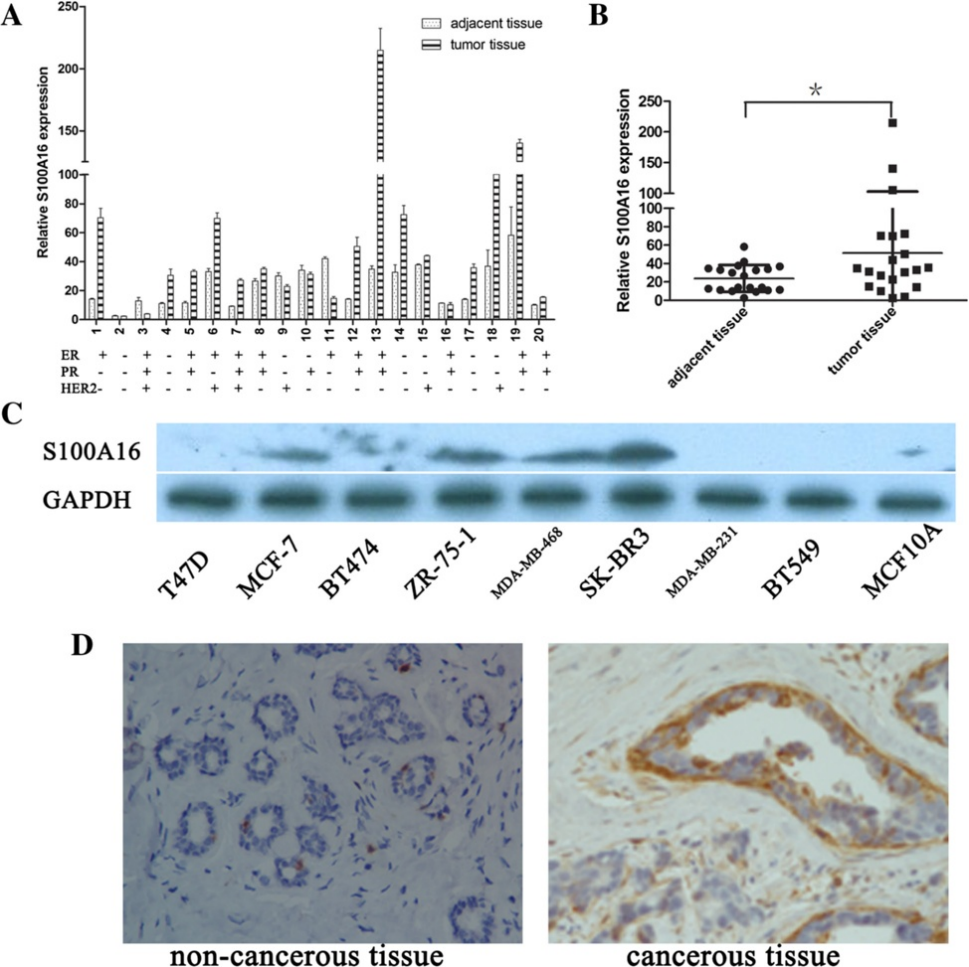
**S100A16 expression in tissues and cell lines. (A)** qRT-PCR analysis of S100A16 expression in 20 pairs of breast cancer tissue and adjacent tissue. Of these 20 pairs of tissues, 14 showed significantly higher S100A16 mRNA expression in the cancer tissue compared with the adjacent tissue (*P* < 0.05). **(B)** A scatter plot showed the mean expression level of S100A16 mRNA in breast cancer tissue was significantly higher than that in adjacent non-cancerous tissue (*P < 0.05 by paired *t*-test). **(C)** Western blot analysis of S100A16 expression in eight breast cancer cell lines and a normal breast cell line MCF10A. It was expressed in MCF10A and five breast cancer lines including MCF-7. **(D)** Representative examples of immunostaining (×400). In non-cancerous tissue, few S100A16 immunostaining was observed, while the expression of S100A16 was significantly higher in cancerous tissue than non-cancerous tissue, especially in the invading front of cancerous tissue.

We measured S100A16 expression in 20 breast cancer tissue samples compared with paired adjacent non-cancerous tissue using qRT-PCR. The clinical characteristics of all subjects are summarized in Table 1. Of these 20 paired samples, 14 showed significantly higher S100A16 mRNA expression in the cancer tissue compared with the adjacent tissue (Figure 1A). There was no significant difference of S100A16 mRNA levels between subgroups of breast cancer with different ER, PR or HER2 status (data not shown). The mean expression level of S100A16 mRNA in breast cancer tissue was also significantly higher than that in adjacent non-cancerous tissue using a scatter plot (Figure 1B). Besides, Immunostainings for S100A16 were performed in breast cancer tissues and the matched non-cancerous tissues. Interestingly, over-expression of S100A16 was observed particularly in the invasive front in breast cancer tissues (Figure 1D), which indicated that S100A16 might be related to EMT. To further study the expression of S100A16 in breast cancer, S100A16 protein expression was detected by Western blot in eight human breast cancer cell lines versus three normal breast epithelial cell lines (MCF10A, 184A1 and 184B5). It was expressed in two ER positive cell lines MCF-7 and ZR-75-1 (ER positive and HER2 negative cell lines) (Figure 1C) and two ER negative cell lines MDA-MB-468 (triple negative cell line) and SK-BR3 (HER2 amplified cell line) (Figure 1C). Additionally, lower expression level of S100A16 was also detected in BT474 (ER positive and HER2 overexpression cell line) and MCF10A cells (Figure 1C). S100A16 protein expression was not detected in other two normal breast epithelial cell lines 184A1 and 184B5 (Additional file 1: Figure S1). Among these limited cell lines, there was no direct correlation between S100A16 and ER levels or HER2 expression although relative levels of S100A16 in MDA-MB-468 and SK-BR3 were higher compared with expressed ER positive cells (Figure 1C).

**Table 1:** **Characteristics of the 20 patients with breast cancer**

**Variables**	**Cases**	
	**No.**	**%**
Ages (mean, range)	56, 37 ~ 70	
≤50	7	35
>50	13	65
Tumor size		
≤2 cm	9	45
>2 cm	11	55
Lymph node involvement		
Negative	11	55
Positive	9	45
Estrogen receptor status		
Negative	8	40
Positive	12	60
Hormone receptor status		
Negative	7	35
Positive	13	65
HER2 status		
Negative	14	70
Positive	6	30
Molecular subtype		
HR+/HER2-	10	50
Triple negative	4	20
HER2+	6	30

HR+, ER and/or PR positive.

### Up-regulation of S100A16 increased the capacities of migration and invasion in MCF-7 and T47D cells

**Figure 2:**
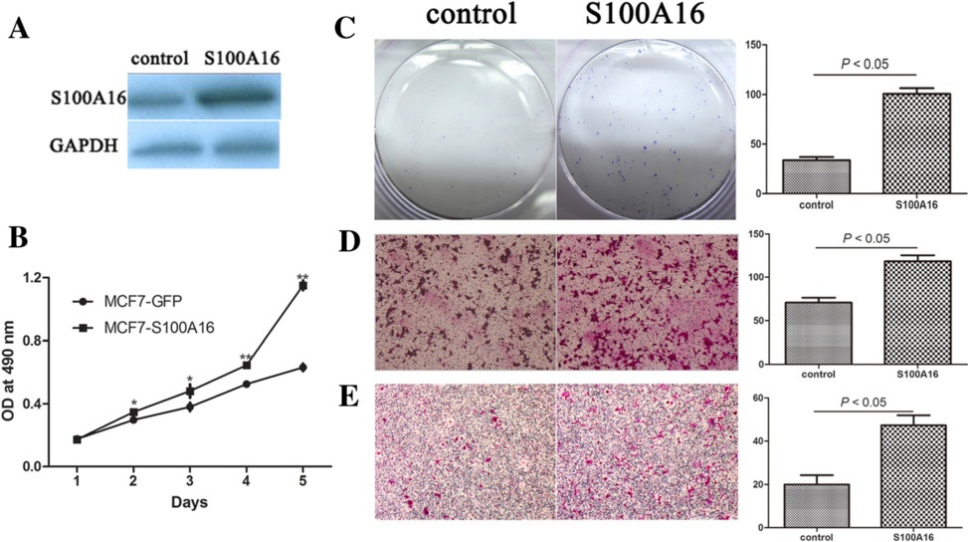
**Up-regulation of S100A16 increased the capacities of proliferation, migration and invasion in MCF-7 cells. (A)** S100A16 was transfected in MCF-7 cells. Western blot was used to measure S100A16 protein expression in control cells (MCF7-GFP) and S100A16 overexpression cells (MCF7-S100A16). **(B)** MTT assay showed that the cell proliferation rate was increased after S100A16 overexpression in MCF-7 cells (Bars, mean ± SD, *P < 0.05, **P < 0.01). **(C)** Colony formation assay confirmed that up-regulation of S100A16 markedly increased the number of cell colonies in MCF-7 cells (*P* < 0.05). **(D, E)** Transwell migration and invasion assays showed that up-regulation of S100A16 increased cell migration **(D)** and invasion **(E)** abilities compared with control cells (*P* < 0.05). Triplicate assays were used for each experiment (Magnification, 10×).

The results of clinical samples indicated that S100A16 may be associated with aggressive behavior in breast cancer (Figure 1A and B). To validate this, we overexpressed S100A16 using pLV-S100A16 lentivirus in MCF-7 and T47D cells. The protein levels of S100A16 were elevated after infection with pLV-S100A16 lentivirus (Figure 2A and Additional file 1: Figure S2). The new cell lines were named as MCF7-S100A16 and T47D-S100A16, and the control cell lines were named as MCF7-GFP and T47D-GFP.

The cell proliferation rate was increased after S100A16 overexpression (Figure 2B). And up-regulation of S100A16 markedly increased the number of MCF-7 cell colonies (Figure 2C). Furthermore, up-regulation of S100A16 increased cell migration (Figure 2D and Additional file 1: Figure S3) and invasion (Figure 2E and Additional file 1: Figure S4) abilities compared with control cells, which were evaluated by specific transwell chambers. All of these results demonstrated that up-regulation of S10016A made cells in an aggressive phenotype.

### Overexpression of S100A16 promoted EMT in MCF-7 and T47D cells

**Figure 3:**
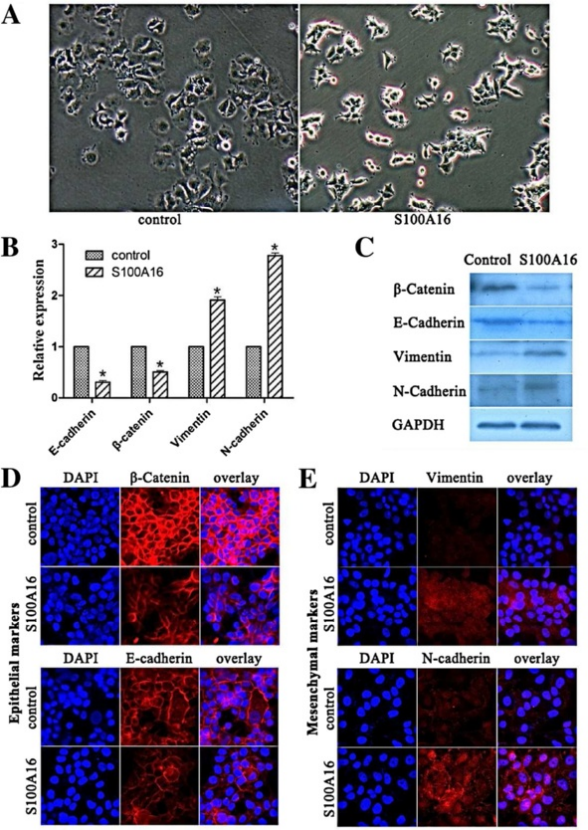
**Overexpression of S100A16 promoted EMT in MCF-7 cells. (A)** Compared with control cells (MCF7-GFP), MCF7-S100A16 cells showed spindle-like, fibroblastic morphology (10×). **(B, C)** qRT-PCR **(B)** and western blot **(C)** analyses showed that epithelial markers E-cadherin and β-Catenin were significantly reduced in mRNA and protein levels in MCF7-S100A16 cells compared with MCF7-GFP cells, and mesenchymal markers Vimentin and N-cadherin were significantly up-regulated in MCF7-S100A16 cells (Bars, mean ± SD, *P* < 0.05). **(D, E)** Immunofluorescence staining was used to examine the location of epithelial and mesenchymal markers. After fixation, the celluar location of E-cadherin (red), β-catenin (red), Vimentin (red) and N-cadherin (red) were analyzed by confocal microscopy. Cell nuclei were stained with DAPI (4′, 6-diamidino-2-phenylindole, blue). Immunofluorescence staining showed that both E-cadherin (red) and β-catenin (red) resided in the cell membrane and intensity of fluorescence were reduced in MCF7-S100A16 cells compared with MCF7-GFP cells **(D)**, whereas the mesenchymal markers Vimentin (red) and N-cadherin (red) were increased by S100A16 **(E)**.

S100A16 over-expressed MCF-7 cells displayed morphologic changes with a spindle-like shape (Figure 3A) (similar changes were founded for T47D-S100A16 cells, data not shown). Up-regulation of S100A16 also disrupted E-cadherin mediated cell-cell adhesion. We observed that epithelial markers E-cadherin and β-Catenin were significantly reduced in mRNA and protein levels in MCF7-S100A16 cells compared with MCF7-GFP cells (Figure 3B and C) and T47D-GFP cells (Additional file 1: Figure S2). In contrast, mesenchymal markers Vimentin and N-cadherin were significantly up-regulated in MCF7-S100A16 cells (Figure 3B and C) and T47D-S100A16 cells (Additional file 1: Figure S2), indicating a characterized feature of EMT.

Consistently, the epithelial and mesenchymal markers were also examined by immunofluorescence staining. Both E-cadherin and β-catenin resided in the cell membrane and the intensity of fluorescence were reduced in MCF7-S100A16 cells compared with MCF7-GFP cells (Figure 3D), whereas the mesenchymal markers Vimentin and N-cadherin were increased by S100A16 (Figure 3E). All of these data suggested that S100A16 was able to repress E-cadherin to promote EMT.

### Up-regulation of S100A16 increased Notch1, ZEB1 and ZEB 2 expression

**Figure 4:**
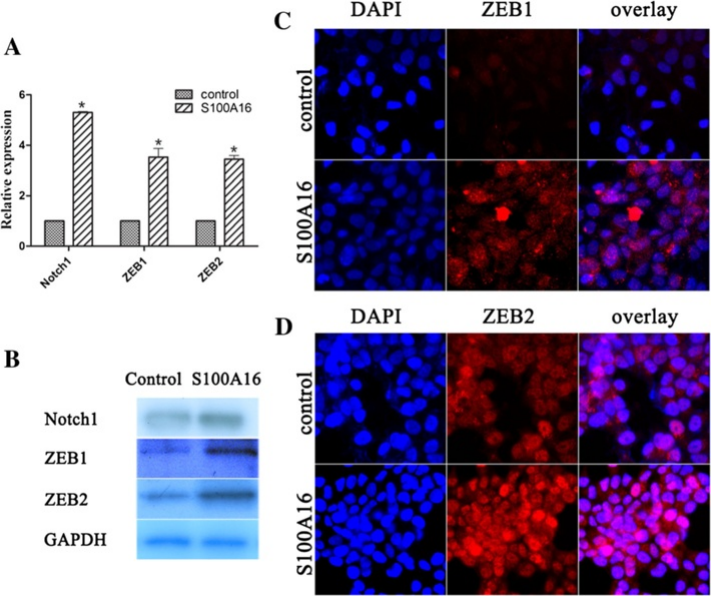
**Up-regulation of S100A16 increased Notch1, ZEB1 and ZEB 2 expression. (A, B)** qRT-PCR **(A)** and western blot **(B)** analyses showed that mRNA and protein levels of Notch 1, ZEB1 and ZEB2 were significantly enhanced in MCF7-S100A16 cells compared with control cells (Bars, mean ± SD, *P* < 0.05). **(C, D)** Immunofluorescence staining was used to examine the location of two transcription factors ZEB1 and ZEB2. After fixation, the celluar location of ZEB1 (red) and ZEB2 (red) were analyzed by confocal microscopy. Cell nuclei were stained with DAPI (4′, 6-diamidino-2-phenylindole, blue). Immunofluorescence staining showed that two transcription factors ZEB1 **(C)** and ZEB2 **(D)** localized in the nucleus and the fluorescence signals were enhanced after S100A16 up-regulation.

It has been reported that Notch signaling pathway is involved in EMT induction [12,13] and Notch1 is a prognosis marker for breast cancer [11]. The transcription factors ZEB1 and ZEB2 are known to repress E-cadherin in various cells [16–18]. To determine whether these transcriptional factors were regulated by S100A16, we observed that Notch1 mRNA and protein levels both were significantly enhanced in MCF7-S100A16 cells (Figure 4A and B) (similar changes were founded for T47D-S100A16 cells, data not shown). Consistently, ZEB1 and ZEB2 in MCF7-S100A16 cells were significantly higher than that in MCF7-GFP cells (Figure 4A and B). Two transcription factors ZEB1 and ZEB2 localized in the nucleus and the fluorescence signals were enhanced after S100A16 up-regulation (Figure 4C and D). These results demonstrated that up-regulation of S100A16 could increase transcription factors to induce EMT.

### Knockdown of Notch1 reversed EMT by decreasing ZEB1 and ZEB 2 expression

**Figure 5:**
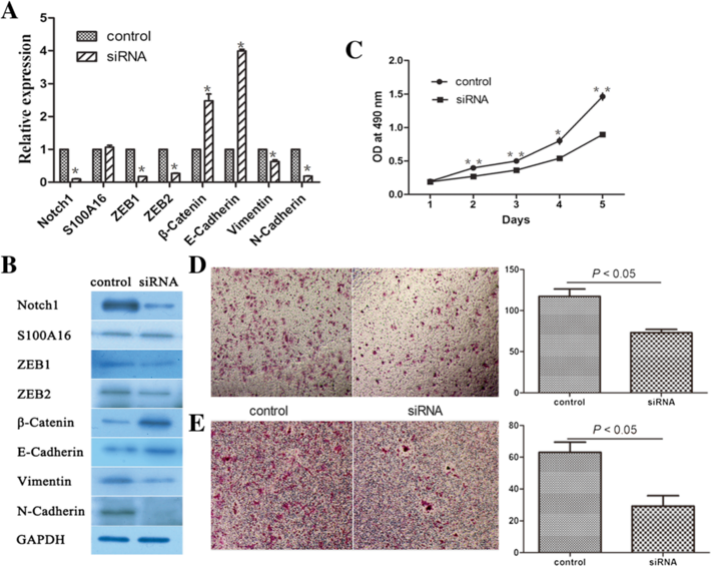
**Knockdown of Notch1 reversed EMT by decreasing ZEB1 and ZEB 2 expression. (A, B)** Specific siRNA was used to knockdown Notch1 in MCF7-S100A16 cells. qRT-PCR (A) and western blot **(B)** analyses showed that Notch1 was successfully knockdown. ZEB1 and ZEB2 were significantly reduced in mRNA and protein levels after knockdown of Notch1. Consequently, epithelial markers E-cadherin and β-Catenin were significantly increased but mesenchymal markers Vimentin and N-cadherin were reduced. There was no variation in the expression of S100A16 influenced by Notch1 knockdown. (Bars, mean ± SD, *P* < 0.05). **(C)** MTT assay showed that knockdown of Notch1 decreased the proliferation of MCF7-S100A16 cells (Bars, mean ± SD, *P < 0.05, **P < 0.01). **(D, E)** Transwell migration and invasion assays showed knockdown of Notch1 decreased migration and invasion in MCF7-S100A16 cells (*P* < 0.05). Triplicate assays were used for each experiment (Magnification, 10×).

As shown in Figure 4A and B, S100A16 had ability to promote EMT through regulation of ZEB1 and ZEB2. Our previous study [19] suggests Notch1 is involved in the induction of EMT in a novel breast cancer mouse model containing human breast and human bone. Here, we hypothesized that S100A16 may promote EMT through Notch1/ZEB signaling pathways. To investigate the role of Notch1, specific siRNA was used to knock down Notch1 in MCF7-S100A16 cells (Figure 5A and B). Transcription factors ZEB1 and ZEB2 were significantly reduced in mRNA and protein levels after knockdown of Notch1 (Figure 5A and B). Consequently, epithelial markers E-cadherin and β-catenin were significantly increased but mesenchymal markers Vimentin and N-cadherin were reduced. There was no variation in the expression of S100A16 influenced by Notch1 knockdown, which indicated that S100A16 was the upstream of Notch1 (Figure 5A and B). Cell proliferation assays showed that knockdown of Notch1 decreased the proliferation of MCF7-S100A16 cells (Figure 5C). Importantly, knockdown of Notch1 decreased migration and invasion in MCF7-S100A16 cells (Figure 5D and E). These data indicated that Notch1 was a major regulator of EMT and it might be an important target site to reduce migration and invasion of breast cancer cells.

### S100A16 activated Notch1 transcriptional activity

**Figure 6:**
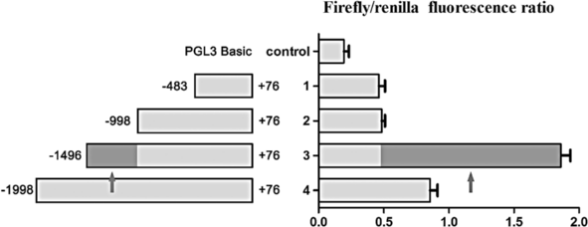
**S100A16 activated Notch1 transcriptional activity.** Dual luciferase reporter assay showed that transcriptional activities were increased in four transfected plasmids in S100A16-transduced MCF-7 cells (Bars, mean ± SD, *P* < 0.05). However, the region from −1496 to +76 had the strongest promoter activity. It seemed that the region from −1496 to −998 was the core part accounting for increasing promoter activity.

Since S100A16 was able to regulate expression of Notch1 (Figure 4A and B), we addressed the question of whether it could affect the transcriptional activity of the Notch1 promoter. In S100A16-transduced MCF-7 cells, transcriptional activities were increased in four transfected plasmids (Figure 6). However, the region from −1496 to +76 had the strongest promoter activity (Figure 6). And it seemed that the region from −1496 to −998 was the core part accounting for increasing promoter activity. This result confirmed that S100A16 could regulate the transcriptional activity of the Notch1 promoter in breast cancer cells.

## Discussion

S100A16 is a novel calcium-binding signaling protein of the EF-hand superfamily [7]. It has been reported that S100A16 is up-regulated in various malignant tumors [5,9], however, the role of S100A16 gene in human breast cancer is yet to be elucidated. In the present study, we first documented here that overexpression of S100A16 occurred in the human breast cancer tumors compared with the paired adjacent normal tissue. This result encouraged us to further investigate the function of S100A16 in the progression of breast cancer. Our previous studies suggest that S100A16 promotes adipogenesis and is involved in weight gain attenuation [7,8]. However, we found that overexpression of the S100A16 gene increased proliferation, migration and invasion in MCF-7 cells, and induced EMT in MCF-7 and T47D cells.

EMT is an invasive phenotype of cancer cells and involved in the events of tumor progression and metastases [10,19]. Multiple transcriptional factors are regulated in the process of EMT, demonstrating differentially in different types of cells. In our cell model, overexpression of S100A16 could up-regulate transcriptional factors ZEB1 and ZEB2, which directly repressed the expression of E-cadherin and enhanced expression of mesenchymal markers E-cadherin and vimentin. All these data indicated that the S100A16 gene might be closely associated with EMT and promote invasion and metastases during the progression of human breast cancer.

We sought to find the mechanisms how the S100A16 regulated ZEB1 and ZEB2 to promote EMT in this cell model. Many studies confirm that ZEB1 and ZEB2 are able to repress E-cadherin in various cells [16–18], and reversal of EMT requires inhibition of ZEB expression [18]. Our recent observation suggests that Notch1 is involved in the induction of EMT in a novel breast cancer mouse model [19]. In agreement with this result, overexpression of S100A16 enhanced the levels of Notch1 and regulated transcriptional activity of Notch1 in breast cancer cells. Knockdown of Notch1 decreased the levels of ZEB1 and ZEB2. Importantly, EMT was reversed after decreasing Notch1 expression in the S100A16 overexpressed cell line. All of these results first indicated that Notch1 was the upstream of ZEB and may play an important role in triggering EMT in breast cancer cells. We first demonstrated here that S100A16 overexpression induced EMT via Notch1/ZEB pathway.

It is well known that EMT is a complicated process with many pathways involving in, such as Wnt pathway, TGF-β pathway, and Shh pathway etc. [10,20–22]. These factors interact with each other to affect the phenotype of cells. Among the tested cell lines, it was interesting to find that S100A16 protein was mainly expressed in epithelial breast cancer cell lines but not in mesenchymal breast cancer cell lines. Moreover, expression of S100A16 was detected in ER-positive cell line, HER2-positive cell line, as well as triple-negative cell line. It is unclear the association among S100A16, ER, and HER2 in breast cancer. More investigation is required to find the relationship of S100A16 with two important clinical biomarkers ER and HER2 in breast cancer. It is also under study to follow up the patients with S100A16 overexpresstion to confirm the function of S100A16 in the progression of breast cancer.

## Conclusions

In summary, the current study demonstrated that overexpression of S100A16 gene promoted EMT via Notch1/ZEB1, ZEB2/EMT pathway in breast cancer cells. These findings suggested that S100A16 could be a target for the intervention of human breast cancer.

## Additional file

Additional file 1: Figure S1. Western blot analyses showed S100A16 protein expression was not detected in normal breast epithelial cells 184A1 and 184B5. **Figure S2.** Western blot analyses showed that epithelial markers E-cadherin and β-Catenin were significantly reduced in protein levels in T47D-S100A16 cells compared with T47D-GFP cells, and mesenchymal markers Vimentin and N-cadherin were significantly up-regulated in T47D-S100A16 cells. **Figure S3.** Transwell migration assay showed that up-regulation of S100A16 increased cell migration abilities in T47D breast cancer cells (P < 0.05). **Figure S4.** Transwell invasion assay showed that up-regulation of S100A16 increased cell invasion abilities in T47D breast cancer cells (P < 0.05).

## Abbreviations

ATCC: American tissue culture collection
EMT: Epithelial-mesenchymal transition
ER: Estrogen receptor
