# Supplementary material for: Up-regulation of S100A16 expression promotes epithelial-mesenchymal transition via Notch1 pathway in breast cancer
Source: J Biomed Sci. 2014 Oct 7;21(1):97. doi: 10.1186/s12929-014-0097-8 (PMC4197258; doi:10.1186/s12929-014-0097-8)
Supplement: Additional file 1: Figure S1. — Western blot analyses showed S100A16 protein expression was not detected in normal breast epithelial cells 184A1 and 184B5. Figure S2. Western blot analyses showed that epithelial markers E-cadherin and β-Catenin were significantly reduced in protein levels in T47D-S100A16 cells compared with T47D-GFP cells, and mesenchymal markers Vimentin and N-cadherin were significantly up-regulated in T47D-S100A16 cells. Figure S3. Transwell migration assay showed that up-regulation of S100A16 increased cell migration abilities in T47D breast cancer cells (P < 0.05). Figure S4. Transwell invasion assay showed that up-regulation of S100A16 increased cell invasion abilities in T47D breast cancer cells (P < 0.05). [file 12929_2014_97_MOESM1_ESM.docx]

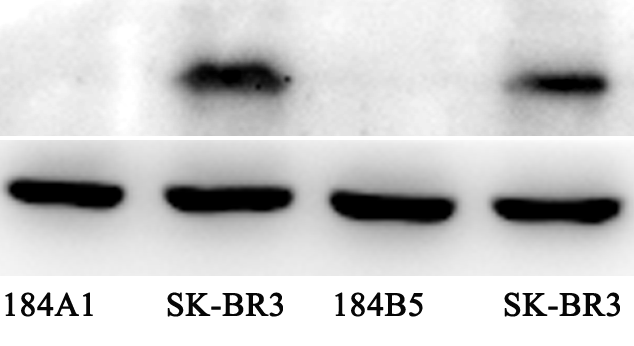


FigS1. Western blot analyses showed S100A16 protein expression was not detected in normal breast epithelial cells 184A1 and 184B5.


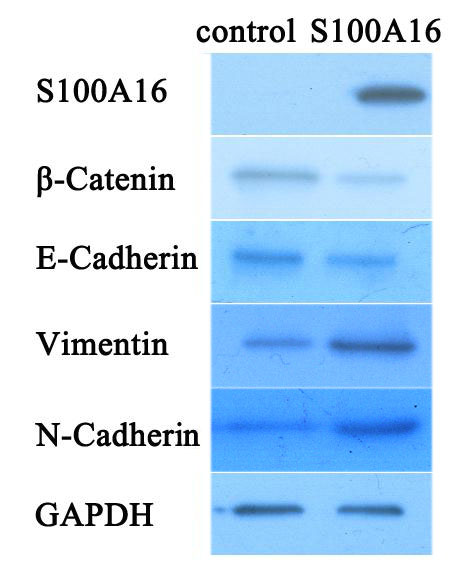


FigS2. Western blot analyses showed that epithelial markers E-cadherin and β-Catenin were significantly reduced in protein levels in T47D-S100A16 cells compared with T47D-GFP cells, and mesenchymal markers Vimentin and N-cadherin were significantly up-regulated in T47D-S100A16 cells.


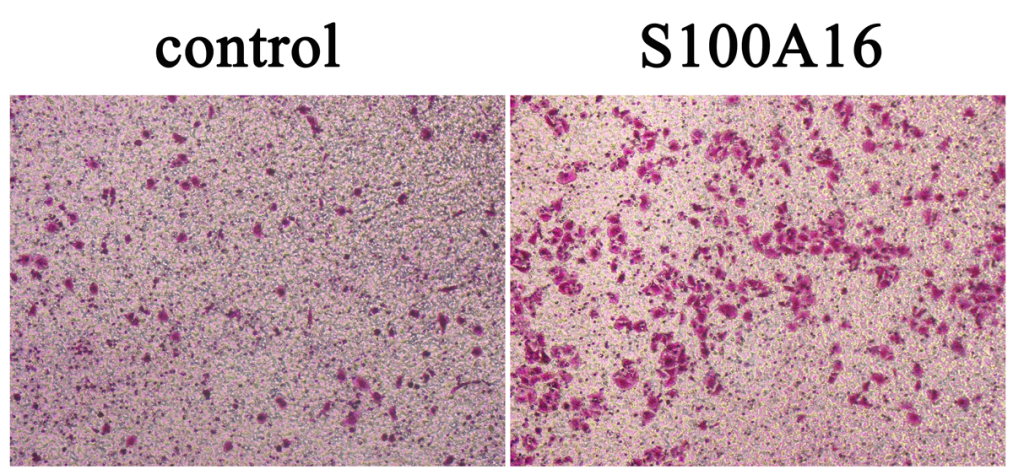


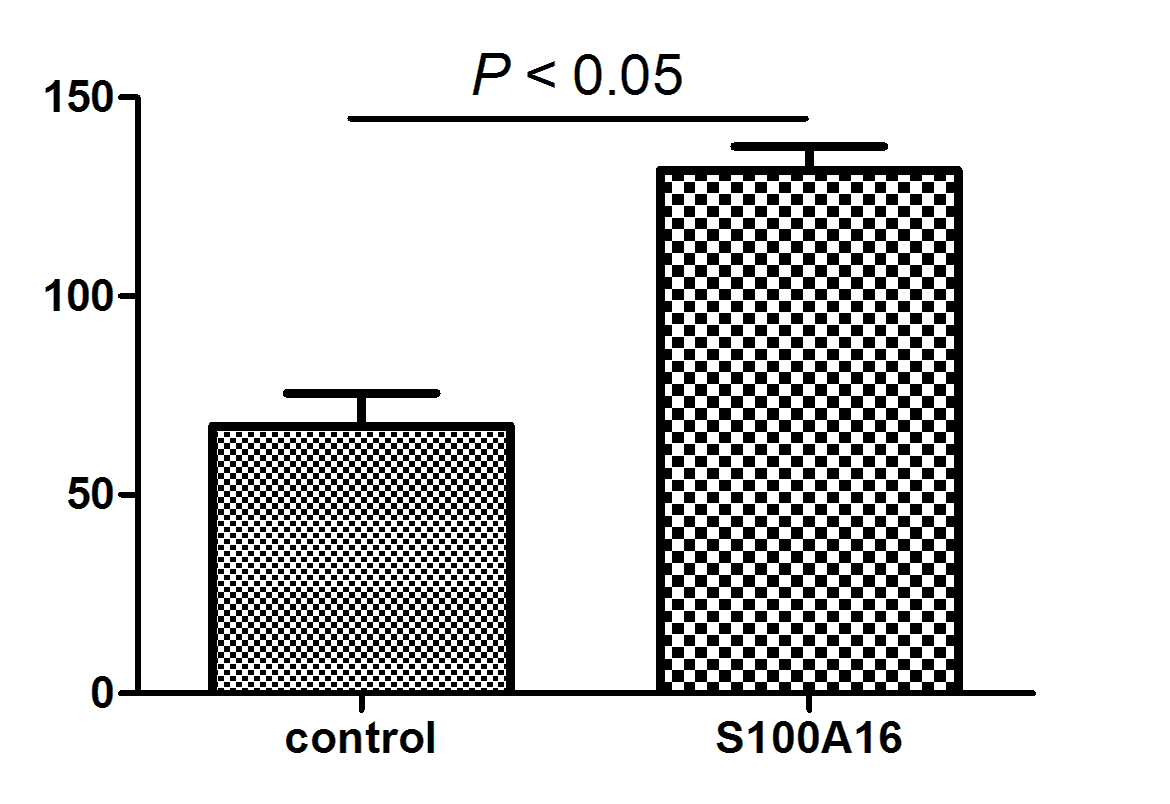


FigS3. Transwell migration assay showed that up-regulation of S100A16 increased cell migration abilities in T47D breast cancer cells (*P* < 0.05).


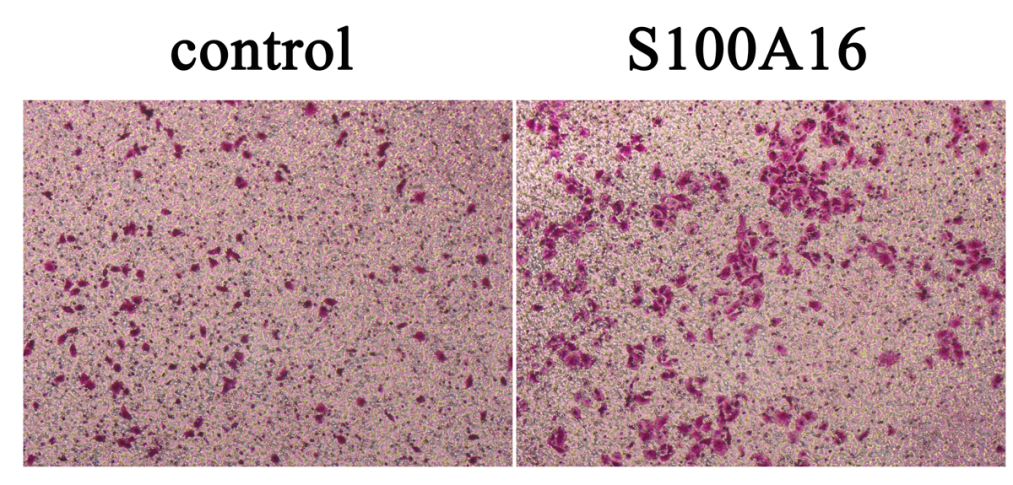


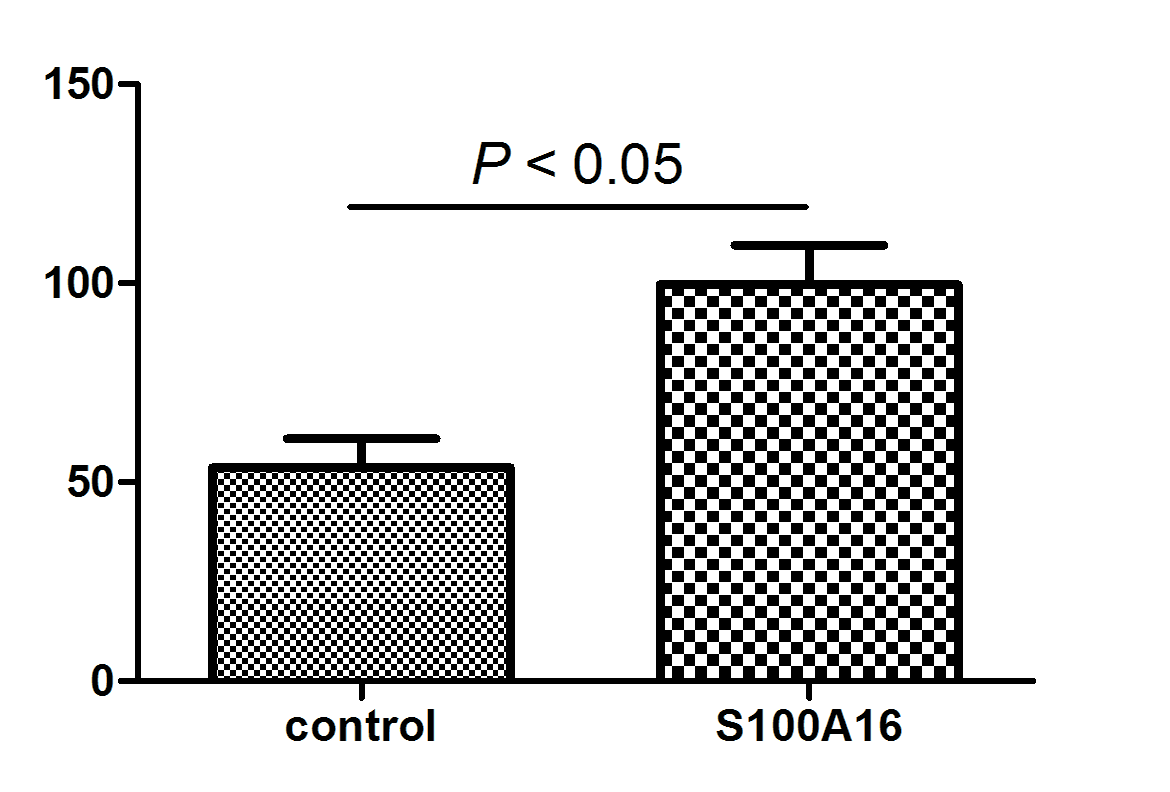


FigS4. Transwell invasion assay showed that up-regulation of S100A16 increased cell invasion abilities in T47D breast cancer cells (*P* < 0.05).
